# The dual role of heme oxygenase in regulating apoptosis in the nervous system of *Drosophila melanogaster*

**DOI:** 10.3389/fphys.2023.1060175

**Published:** 2023-02-13

**Authors:** Terence Al L. Abaquita, Milena Damulewicz, Grzegorz Tylko, Elżbieta Pyza

**Affiliations:** Department of Cell Biology and Imaging, Institute of Zoology and Biomedical Research, Faculty of Biology, Jagiellonian University, Cracow, Poland

**Keywords:** neuroprotection, neurotoxicity, aging, autophagy, oxidative stress

## Abstract

Accumulating evidence from mammalian studies suggests the dual-faced character of heme oxygenase (HO) in oxidative stress-dependent neurodegeneration. The present study aimed to investigate both neuroprotective and neurotoxic effects of heme oxygenase after the *ho* gene chronic overexpression or silencing in neurons of *Drosophila melanogaster*. Our results showed early deaths and behavioral defects after pan-neuronal *ho* overexpression, while survival and climbing in a strain with pan-neuronal *ho* silencing were similar over time with its parental controls. We also found that HO can be pro-apoptotic or anti-apoptotic under different conditions. In young (7-day-old) flies, both the cell death activator gene (*hid*) expression and the initiator caspase Dronc activity increased in heads of flies when *ho* expression was changed. In addition, various expression levels of *ho* produced cell-specific degeneration. Dopaminergic (DA) neurons and retina photoreceptors are particularly vulnerable to changes in *ho* expression. In older (30-day-old) flies, we did not detect any further increase in *hid* expression or enhanced degeneration, however, we still observed high activity of the initiator caspase. In addition, we used curcumin to further show the involvement of neuronal HO in the regulation of apoptosis. Under normal conditions, curcumin induced both the expression of *ho* and *hid*, which was reversed after exposure to high-temperature stress and when supplemented in flies with *ho* silencing. These results indicate that neuronal HO regulates apoptosis and this process depends on *ho* expression level, age of flies, and cell type.

## 1 Introduction

Heme oxygenase (HO) has been reported to have a multifunctional role in cellular homeostasis. Its key function is the degradation of heme, which is the iron-protoporphyrin complex with important functions in biological systems. Free intracellular heme has deleterious effects which are pro-oxidant in nature and can damage various cell organelles. In effect, the regulation of heme level reflects a cytoprotective role of HO (Yoshida and Migita, 2000; Kikuchi et al., 2005; Kumar and Bandyopadhyay, 2005). In mammals, two isoforms of HO have been identified, namely: HO-1 and HO-2. A third member, HO-3, has also been described but it is encoded by a pseudogene (splice-variant of HO-2) which is specific to rats (McCoubrey et al., 1997; Scapagnini et al., 2002; Hayashi et al., 2004). HO-1 is a highly dynamic inducible form that can be upregulated by a variety of environmental stimuli and toxicants (Dennery, 2000; Schipper, 2000; Zhang et al., 2006; Kim et al., 2008), which is an adaptive mechanism of cellular protection from oxidative damage during stress (Poss and Tonegawa, 1997b). Another role of HO-1 in physiological and pathological contexts has been shown in development (Zhao et al., 2009) as well as in iron mobilization and distribution (Poss and Tonegawa, 1997a; Kovtunovych et al., 2010; Radhakrishnan et al., 2011). The second, constitutive form HO-2 (Muñoz-Sánchez and Chánez-Cárdenas, 2014) is important for the proper functioning of some tissues, including brain (Ewing & Maines, 1997; Boehning and Snyder, 2003; Mancuso, 2004). The HO-dependent system is known to be involved in many cellular processes, such as oxidative stress, inflammation and apoptosis, which are regulated by heme degradation products (carbon monoxide, ferrous ions and biliverdin).

In *Drosophila melanogaster*, HO is encoded by a single gene (*ho*) (Zhang et al., 2004). *Drosophila* HO (dHO) has been extensively studied in the retina where it plays a role in viability, development, iron accumulation, cell death (Cui et al., 2008), signaling of DNA damage (Ida et al., 2013), protection against DNA damage caused by UV and white light (Damulewicz et al., 2017a; Damulewicz et al., 2017b), phototransduction, DNA repair, immune responses (Damulewicz et al., 2019), and circadian clock (Damulewicz et al., 2017a; Klemz et al., 2017; Damulewicz et al., 2019). dHO is an inducible form and its expression can be enhanced in the brain by both paraquat (a pro-oxidant compound) and curcumin (an anti-oxidant substance). Elevated *ho* mRNA level by pro- or anti-oxidative compounds is accompanied by changes in the expression pattern of genes involved in autophagy (degradation and recycling process) and apoptosis (elimination of damaged cells). Moreover, increasing or decreasing *ho* expression in neurons affects the transcript levels of these genes (Abaquita et al., 2021).

The antioxidative role of HO is based on scavenging of free radical species, particularly in the brain and rest of the nervous system in both mammals and *D. melanogaster* (Loboda et al., 2008; Abaquita et al., 2021). The energy-demanding synaptic transmission (Harris et al., 2012) is usually associated with the overproduction of reactive oxygen and nitrogen species (e.g. ROS and RNS). If they are left at high levels, neurons and glial cells undergo oxidative stress, along with detrimental modifications in proteins, lipids, and nucleic acids (Cerutti, 1985; Cross et al., 1987; Pero et al., 1990). Therefore, the nervous system requires a sustained upregulation of HO-1 in order to maintain a balanced redox status in neurons and glial cells for their normal functions. However, some studies have reported that constant overexpression of HO-1 leads to the development of aging-related neurodegenerative and neuroinflammatory disorders as well as to major neurodevelopmental diseases (Schipper et al., 2009; Barone et al., 2014; Schipper et al., 2019; Wu & Hsieh, 2022). The elevated HO-1 level can stimulate intracellular oxidative stress, iron sequestration, mitochondrial damage, and macro-autophagy (mitophagy), which are the core neuropathological tetrad features of a variety of disorders mentioned above (Schipper et al., 2009; Barone et al., 2014; Schipper et al., 2019; Wu & Hsieh, 2022). Moreover, it has been shown that HO proteins can translocate to non-endoplasmic reticulum compartments (Lin et al., 2007; Sacca et al., 2007; Bindu et al., 2011; Bolisetty et al., 2013; Bansal et al., 2014; Biswas et al., 2014; Dunn et al., 2014; Tibullo et al., 2016; Mascaró et al., 2021). The catabolites of heme degradation can also diffuse outside of the cell (Chiang et al., 2018; Luu Hoang et al., 2021; Yang & Wang, 2022), which may extrinsically modulate other biological processes, including the toxic ones. This sinister role of HO-1 was recognized after analysing the effects of HO-1 overexpression in the brain (specifically in astrocytes), dissecting the HO pathway and its regulatory elements (Schipper et al., 2009; Barone et al., 2014; Nitti et al., 2018; Waza et al., 2018; Schipper et al., 2019; Luu Hoang et al., 2021; Wu & Hsieh, 2022), and studying a possibility that HO invades non-target cell compartments (Bansal et al., 2014; Mascaró et al., 2021; Yang & Wang, 2022).

It means that the constant high level of HO-1 is therefore associated with both cytoprotective and cytotoxic functions in many mammalian and cell-based model systems, especially in studying oxidative stress-dependent neurodegeneration. These biological functions of HO, which denote its dual-faced character, might be context dependent and are assumed to vary according to many conditions, such as the expression level, age, and cell types. Using *D. melanogaster* as a model, we exploited the GAL4/UAS system to examine the effects of overexpression or partial silencing of *ho* expression in neurons. We aimed to check age-dependent changes in the survival and climbing ability of adult flies with different *ho* expression levels in neurons. Then, we employed different conditions (i.e. different *ho* expression levels and aging) to further investigate how neuronal HO affects the regulation of apoptosis and autophagy, which we assumed to be two of the many mechanisms that are regulated by *ho* expression levels in the *Drosophila* brain (Damulewicz et al., 2019; Abaquita et al., 2021). We targeted the expression of *ho* in two specific cell types (i.e. dopaminergic neurons and eye photoreceptors) in order to visualize degeneration of specific neurons, in young and old flies, which we hypothesized to vary depending on cell types. In addition, we incorporated curcumin into our model system as it has been shown to affect *ho* expression in the brain and the genes related to apoptosis and autophagy (Abaquita et al., 2021), as well as adult longevity and behavior (Lee et al., 2010; Phom et al., 2014; Akinyemi et al., 2018; Chen et al., 2018). Heat stress was also exploited to check whether the previously described effects of curcumin are temperature-dependent in order to verify the extent of its neuroprotective potential. Our data showed that neuronal HO can contribute positively or negatively to the regulation of apoptosis depending on its gene expression level, cell types, and age of flies. It seems that dHO in neurons has a stronger effect on apoptosis than on autophagy. Moreover, we showed that HO is responsible for the pro-apoptotic potential of chronic curcumin supplementation.

## 2 Materials and methods

### 2.1 Animals

The following strains of *D. melanogaster* were used: wild-type Canton S (CS); *elav-Gal4* (a strain that expresses *Gal4* under the control of the *embryonic lethal abnormal vision* [*elav*] promoter; pan-neuronal cell marker); *GMR-Gal4* (*Gal4* under the control of the *Glass Multiple Receptor* [*GMR*] enhancer-promoter, predominantly in the retina) (a kind gift along with *elav*-*Gal4* from Dr. R. Stanewsky, Institute of Neuro- and Behavioral Biology, University of Münster, Germany); *ple*-*Gal4* (*Gal4* under the control of the *pale* [*ple*] promoter; dopaminergic cell marker); *UAS*-*hoRNAi* (a strain which expresses dsRNA for the *ho* gene under the control of UAS sequence) (Cui et al., 2008); *UAS*-*ho* (expresses *ho* under the control of UAS sequence) (Cui et al., 2008); and *UAS*-*Valium10* (control for *hoRNAi* strain).

The GAL4/UAS system was used to generate flies with *ho* overexpression or silencing in all neurons (*elav* > *ho* or *elav > hoRNAi*, respectively). Those of *elav* > CS and CS > *ho* were used as parental controls for pan-neuronal *ho* overexpression, whereas, *elav* > *Valium10* and CS > *hoRNAi* were control groups for pan-neuronal *ho* silencing. Prior to the conduct of the study, we confirmed that the parental lines had a normal level of *ho* gene expression (Supplementary Figure S1A), and in both genotypes *elav* > *ho* and *elav* > *hoRNAi* the level of protein was changed (Supplementary Figure S1B). These genotypes were used to study the influence of different pan-neuronal *ho* expression on survival and climbing behavior, which then followed further investigation on apoptosis at the transcriptional level and enzyme activity in two different age groups (7- and 30-day-old).

All flies were maintained under 12 h of light followed by 12 h of darkness (LD12:12).

### 2.2 Survival and climbing assays

Flies with pan-neuronal *ho* overexpression or silencing, along with their controls, were firstly examined for survival and in climbing assays to check the influence of different neuronal *ho* mRNA levels on adult longevity and behavior. Each assay consisted of 30 males for each genotype (repeated at least three times). Survival assay was performed by counting every day the number of dead flies. For the climbing assay, flies were transferred to an empty vial and after a short recovery, they were gently tapped to the bottom of the vial. After 15 s, individuals that climbed vertically beyond the 5-cm marked line were counted. The climbing assay was carried out at ZT1 in dim red light under constant conditions. This assay was carried out in 7-, 14-, 30-, and 60-day-old flies.

### 2.3 RNA isolation, cDNA synthesis, and quantitative PCR

Male flies for each genotype were collected at ZT16 (Zeitgeber Time, ZT0 indicates lights-on and ZT12 lights-off). Heads were isolated and subjected to total RNA isolation using TriReagent (MRC Inc., Irvine, CA, United States) according to the manufacturer’s protocol. Brains were extracted from fixed heads at 100% ethanol for 2 h. The RNA quality and quantity were assessed using Nanodrop 2000 (Thermo Fisher Scientific, MA, United States). cDNA was synthesized using a High Capacity cDNA Reverse Transcription Kit (Thermo Fisher Scientific, Vilnus, Lithuania) with random primers according to the provider’s instruction. Gene expression was examined using StepOnePlus Real-Time PCR System and SYBR Green Master Mix (KAPA Biosystems, Cape Town, South Africa) in the presence of specific primer sequences (the specificity was controlled with Primer-BLAST and gel electrophoresis) for the target genes which are listed in Table 1. This entire procedure was followed in experiments analysing gene expression levels, which were repeated at least three times per genotype with approximately 20 fly heads for each replicate.

**TABLE 1:** Primer sequences of the genes which were used in this study.

Gene		Sequence	Accession number
ho	F	5′-ACC​ATT​TGC​CCG​CCG​GGA​TG-3′	CG14716
	R	5′-AGT​GCG​ACG​GCC​AGC​TTC​CT-3′	
rpl32	F	5′-TAT​GCT​AAG​CTG​TCG​CAC​AAA​TG-3′	CG7939
	R	5′-AGC​ACG​TGT​ATA​AAA​AGT​GCC​A-3′	
hid	F	5′-CAT​CCA​TGG​CCA​CAT​CAG​T-3′	CG5123
	R	5′-TTA​CAC​GTC​TCC​TGC​GCT​TT-3′	
atg5	F	5′-GAC​ATG​CTC​GTC​AAG​CTC​AA-3′	CG1643
	R	5′-TCC​ATT​AGC​CTC​CGA​TTG​AC-3′	
atg10	F	5′-TCA​GAC​CCT​TTA​TGG​CAT​TG -3′	CG12821
	R	5′-GGC​TTT​CCG​AAA​CTG​CTT​TAG-3′	

### 2.4 Estimation of caspase Dronc activity

The activity of caspase Dronc was measured using Caspase-Glo 9 Assay (Promega, United States). Male flies, 7- or 30-day-old, were decapitated at ZT16. Heads were collected and then lysed for 30 min at 4 °C with an HBSS buffer, pH 7.9, supplemented with Na_2_EDTA (0.1 mM, POCH, Poland), EGTA (0.1 mM, Sigma, Germany), and 2% NP-40. The amount of the extraction buffer used for cell lysis followed a 1:1 ratio of the number of heads and the volume of the extraction buffer (1 μl per head). Then, the samples were centrifuged at 13,200x*g* for 1 h at 4°C. The supernatant was collected and protein content was estimated by Nanodrop 2000. Protein solutions were then mixed with the Caspase-Glo^®^ 9 reagent at a 1:1 ratio, according to the manufacturer’s protocol, to individual wells in a 96-well plate. After 35 min of incubation at room temperature in darkness, the Relative Light Unit (RLU) of each sample was measured using the end-point, luminescence (LUM) read mode function of a plate reader (SpectraMax iD3, Molecular Devices, San Jose, CA, United States). Whole-head homogenates were used for this experiment with at least three repetitions per genotype and at least 40 fly heads for each replicate. This headcount was initially calibrated with the total protein content that has been tested to detect caspase activity using cells from *in vitro* culture.

### 2.5 Brain immunostaining

To check the effect of *ho* expression level on dopaminergic cell degeneration, *ple > ho* or *ple > hoRNAi* were used. The parental control group was *ple >* CS.

Heads of 7- or 30-day-old males for each genotype were fixed at ZT1 in 4% paraformaldehyde (PFA) in phosphate buffer saline (PBS) for 1 h at room temperature (RT). Then, brains were isolated and washed in PBS for 10 min and three times in 0.2% phosphate buffer saline with Triton X-100 (PBST) for 10 min each. Next, they were incubated in 5% normal goat serum (NGS) in 0.2% PBST for 45 min at RT. Subsequently, brains were incubated for 3 days at 4°C with mouse primary anti-Tyrosine Hydroxylase (1:1000, ImmunoStar) serum. Thereafter, brains were washed three times in 0.2% PBST for 10 min each and incubated for 2 h at RT with secondary goat anti-mouse Cy3-conjugated (1:250, Abcam) antibody. Finally, brains were washed three times in 0.2% PBST and once in PBS for 10 min each before mounting them in a Vectashield medium (Vector). Z-stacks of the whole-mount brains were obtained with a Zeiss Meta 510 Laser Scanning Microscope. Direct counting of DA neurons was done in each of the five DA clusters, namely: PAL (protocerebral anterior lateral), PPL1 (posterior inferiorlateral protocerebrum), PPL2 (posterior lateral protocerebum), PPM1/2 (posterior superiormedial protocerebrum and posterior inferiormedial protocerebrum), and PPM3 (superior posterior slope) (Mao and Davis, 2009), using ImageJ software. This experiment was repeated three times per genotype with approximately 10 heads for each replicate.

### 2.6 Scanning electron microscopy

To visualize degeneration in the external morphology of the fly’s eye, we used scanning electron microscopy (SEM) and flies with *ho* overexpression or silencing in the retina (GMR > *ho* or GMR > *hoRNAi*, respectively). The experimental groups were compared to the parental control group GMR > CS. Heads of 7- or 30-day-old adult males were fixed at ZT1 in glutaraldehyde (2.5%) in cacodylate buffer overnight and washed four times, 15 min each, with cacodylate buffer on the next day. Subsequently, heads were dehydrated in graded ethanol series (15%–100%), which was then followed by pure acetone. They were dried in CO_2_ at a critical point and coated with gold. Next, they were examined and photographed with a JEOL JSM5410 scanning electron microscopy.

### 2.7 Histology

To visualize degeneration of photoreceptors in the retina, we used a histological staining of GMR > *ho,* GMR > *hoRNAi* and the control GMR > CS. Male flies, 7- or 30-day-old, were decapitated at ZT1 and the heads were fixed in 2% glutaraldehyde and 2.5% paraformaldehyde in a cacodyl buffer with CaCl_2_ for 1 h, and postfixed for 1 h in 2% OsO_4_ in a veronal acetate buffer with CaCl_2_ and sucrose. Next, samples were dehydrated in an alcohol series and propylene oxide and embedded in Poly/Bed 812 (Polysciences) resin. Serial sections of 1 µm were cut and stained with a mixture of methylene blue, azure II, and borate solution before mounting with Permount. Images were obtained with Zeiss light microscope.

### 2.8 Curcumin feeding experiments

Males were fed for 14 days with a standard diet (yeast-cornmeal-agar) supplemented with curcumin (EMD Millipore Corp., Darmstadt, Germany) which was dissolved in 1% ethanol and mixed at 1 mg/mL of the medium according to methods described in our previous study (Abaquita et al., 2021).

Curcumin feeding experiments were divided into two steps. First, we checked the effects of curcumin on survival and climbing ability of CS flies during 14 days when they were maintained at 25°C or 29°C and on three different diets, namely: standard diet mixed with curcumin dissolved in 1% ethanol; standard diet without curcumin but with 1% ethanol; and standard diet only. Then, the following gene expression; *ho*, *atg5*, and *hid* was examined in brains of the treated and non-treated flies. Before this experiment with curcumin exposure, flies were adapted to the normal temperature (25°C) and a standard diet for 3 days. Both survival and climbing assays were repeated three times (30 flies in each treatment), while gene expression analysis was done for at least three repetitions in each treatment with 25 brains per repetition.

In the second step of experiments curcumin supplementation was administered for 14 days to *elav* > *ho* and *elav* > *hoRNAi*, along with their respective parental controls. Flies of different genotypes that were not treated with curcumin (only 1% ethanol in standard diet) were regarded as controls. After the treatment survival and climbing ability of experimental and control flies were examined. Then, heads were decapitated at ZT16 in order to examine *hid* expression levels. This step was repeated at least three times for each genotype with 30 flies per repetition.

### 2.9 Statistical analysis

Differences in gene expression between three genotypes for pan-neuronal *ho* overexpression and silencing in each age group (7- or 30-day-old), and after feeding with or without curcumin, were statistically analysed using the non-parametric analysis of variance (ANOVA)—the Kruskal–Wallis test and *post hoc* Conover-Iman’s test. The non-parametric Mann-Whitney test was then used to compare between two different age groups or other treatments (i.e. thermal and curcumin feeding) for each genotype. This combination of statistical tests was also utilized in detecting differences in all survival and climbing data as well as in comparing three different treatment groups of the curcumin feeding experiment in each temperature condition.

The Mann-Whitney test was also used to compare the caspase Dronc activity between experimental and control genotypes and then between different age groups per genotype. Unpaired *t-*test with Welch’s correction was exploited for comparing two different genotypes and aging effects in DA neuron degeneration. Descriptive statistics was used for evaluation of photoreceptor degeneration (i.e. the eye external morphology and the retina structure).

All data analyses were performed with the R/R Studio freeware statistical package version 4.2.0 (http://www.R-project.org/) or GraphPad Prism 7.05 software.

## 3 Results

### 3.1 Physiological *ho* level in neurons is important for the survival and climbing behavior of adult fruit flies

**FIGURE 1:**
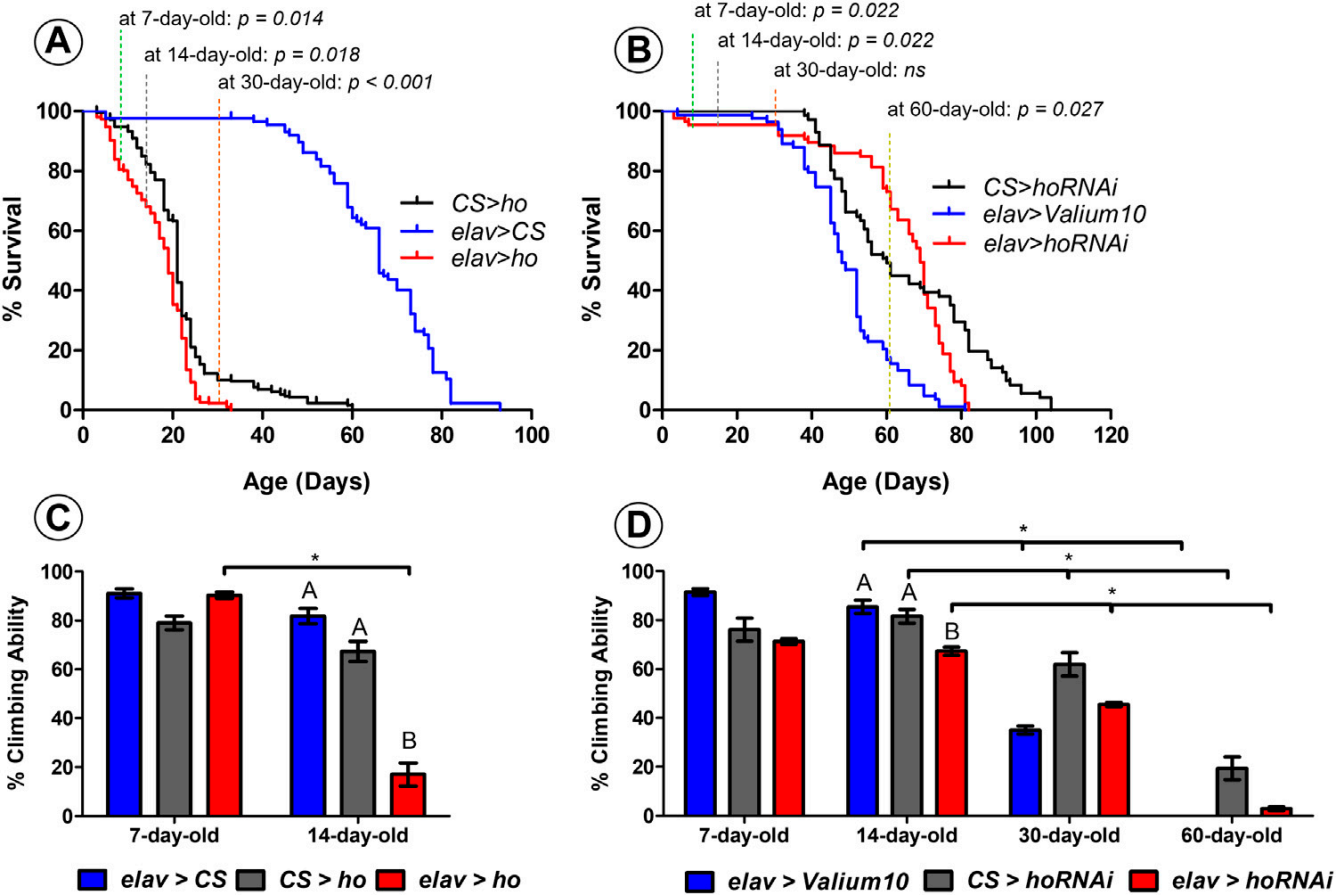
Neuronal *ho* expression level is important for the survival and climbing ability of *Drosophila*. Percentage of survival for flies with pan-neuronal *ho* overexpression **(A)** was significantly different than parental controls in 7-, 14-, and 30- day-old flies (non-parametric ANOVA Kruskal–Wallis and *post hoc* Conover-Iman tests; *p* values are indicated); whereas, for pan-neuronal *ho* silencing **(B)**, differences in percentage of survival were statistically significant in 7-, 14-, and 60- day-old flies (non-parametric ANOVA Kruskal–Wallis and *post hoc* Conover-Iman tests; *p* values are indicated). For percentage of climbing ability which is shown as means ± SEM **(C–D)**, statistically significant differences between genotypes are indicated with different *letters* (after 14 days for *ho* overexpression and *ho* silencing as well; non-parametric ANOVA Kruskal–Wallis and *post hoc* Conover-Iman tests: *ho* overexpression *p* = 0.024, *ho* silencing *p* = 0.050) and *asterisks* between different age groups (non-parametric Mann-Whitney test: 7 days vs. 14 days for *elav* > *ho p* = 0.050, 14 days vs. 30 days for *elav* > *Valium10 p* = 0.050, CS > *hoRNAi p* = 0.050*, elav* > *hoRNAi p* = 0.038; 14 days vs. 60 days for *elav* > *Valium10 p* = 0.032, CS > *hoRNAi p* = 0.050*, elav* > *hoRNAi p* = 0.050). Each assay was repeated at least three times with 30 flies per genotype in each repetition.

Both overexpression and silencing of *ho* in neurons caused changes in percentage of survival and climbing ability of adult flies over time (Figure 1).

Early death phenotype (Figure 1A) and climbing defects (Figure 1B) were observed in flies with pan-neuronal *ho* overexpression. After 7 days, *elav* > *ho* showed 84% of survival which was significantly different comparing with both control groups (95% and 98% for CS > *ho* and *elav* > CS, respectively). After 14 days from eclosion, percentage of survival flies was lower in the experimental group (*elav* > *ho*: 68%) compared to the control ones (CS > *ho*: 82% and *elav* > CS: 98%). After 30 days, there were only 2% of survived *elav* > *ho* flies, while 10% and 98% in case of CS > *ho* and *elav* > CS, respectively. A similar survival pattern was detected between CS > *ho* and *elav* > *ho* after 14 and 30 days. Nevertheless, *elav* > *ho* flies survived no more than 33 days, while CS > *ho* and *elav* > CS lived for 60 and 93 days, respectively. Although the differences between *elav > ho* and control lines CS > *ho* and *elav* > CS in survival were significant, it is likely that other factors, such as genetic background of *elav > ho*, might affect longevity because in middle age flies the survival of *elav > ho* was similar to *CS > ho*. This does not explain, however, why in younger and older flies the difference was larger between *elav > ho* and *CS > ho*.

In case of climbing ability there were no statistically significant the differences between *elav* > *ho* and its parental controls after 7 days. We found, however, that more than 80% of the experimental flies at the age of 14 days were not able to climb over the 5-cm limit, which was significantly different from control groups (less than 30% did not pass the indicated limit). In addition, age-dependent decrease in climbing was only observed in *elav* > *ho* and not among control groups. We did not perform the climbing assay after 30 days because only one to two *elav* > *ho* flies survived until this age.

For flies with pan-neuronal *ho* silencing, significant differences were observed in young flies (7–14 days: 97% survival for *elav* > *hoRNAi*, 100% for CS > *hoRNAi* and 99% for *elav* > *Valium10*) and old ones (60 days: 73% for *elav* > *hoRNAi*, 49% for CS > *hoRNAi* and 17% for *elav* > *Valium10*) (Figure 1C). Surprisingly, we did not observe changes in survival in 30 days old flies. Flies with pan-neuronal *ho* silencing survived 82 days, while 81 days lived *elav* > *Valium10* and 104 days CS > *hoRNAi*. Regarding climbing behavior, significant differences between experimental and control groups were found only after 14 days (Figure 1D). In addition, age-dependent decrease in the climbing ability was observed for all genotypes when compared between 14-, 30- and 60-day-old flies.

Overall, these results suggest that any modification in the expression level of *ho* in neurons leads to age-specific differences in the percentage of survival and climbing ability of adult flies. This indicates that both apoptosis and autophagy might be involved in the observed changes. It is also interesting to know whether these processes are also influenced by age as we found age-dependent effects on survival and climbing in flies with continuous overexpression or partial silencing of *ho* in neurons.

### 3.2 Age-dependent induction of the cell death activator *hid* gene and caspase Dronc activity by *ho* expression in neurons

**FIGURE 2:**
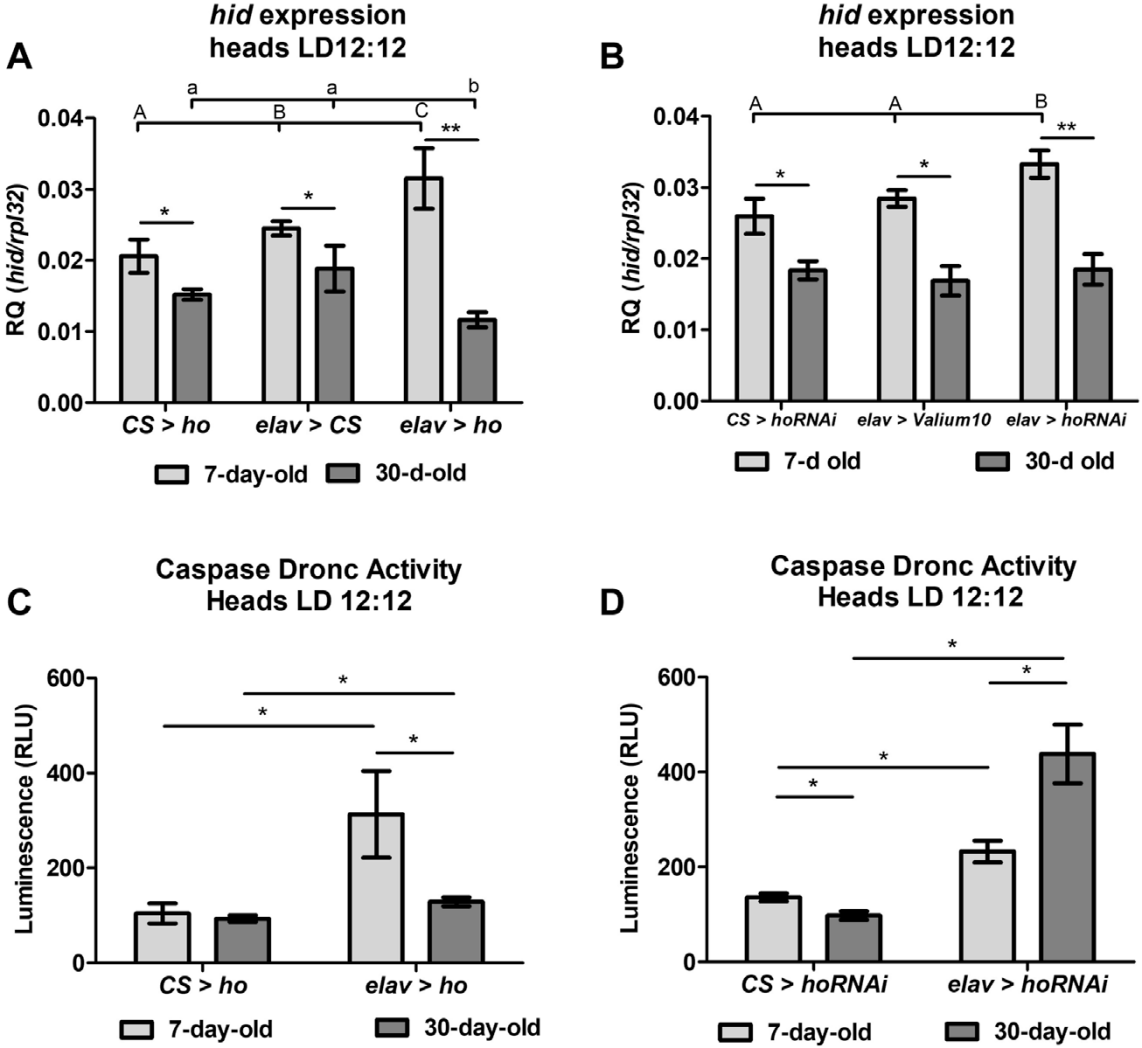
Aging affects the cell death activator *head involution defective* (*hid*) gene expression and the caspase Dronc activity in heads of flies with pan-neuronal *ho* overexpression or silencing. For *hid* expression **(A,B)**, means ± SD), statistically significant differences between genotypes are indicated with different *letters* (capital letters for 7-day-old and small letters for 30-day-old flies; non-parametric ANOVA Kruskal–Wallis and *post hoc* Conover-Iman tests: *ho* overexpression in 7-day-old *p* = 0.008, 30-day-old *p* = 0.006; *ho* silencing in 7-day-old *p* = 0.008, 30-day-old *p* = 0.6) and between different age groups by *asterisks* (7-day-old vs. 30-day-old in each genotype; non-parametric Mann-Whitney test: *elav* > *ho p* = 0.008; CS > *ho p* = 0.03; *elav* > CS *p* = 0.02; *elav* > *hoRNAi p* = 0.004; CS > *hoRNAi p* = 0.04; *elav* > *Valium10 p* = 0.04). For caspase Dronc activity **(C,D)**, means ± SEM), statistically significant differences between genotypes and between age are indicated with *asterisks* (non-parametric Mann-Whitney test: *ho* overexpression in 7-day-old *p* = 0.03, 30-day-old *p* = 0.05; *ho* silencing in 7-day-old *p* = 0.03, 30-day-old *p* = 0.03; 7-day-old vs. 30-day-old in each genotype through non-parametric Mann-Whitney test: *elav* > *ho p* = 0.03, *elav* > *hoRNAi p* = 0.03, CS > *ho p* = 1.00, CS > *hoRNAi p* = 0.03). Both experiments were repeated at least three times per genotype or age group (*hid* expression: 20 heads per repetition, caspase Dronc activity: 40 heads per repetition).

Aging caused a general decline of *hid* mRNA between 7- and 30-day-old flies (Figures 2A, B). Moreover, statistically significant differences in *hid* mRNA level in whole head homogenates was detected between young and older flies with both overexpressed (Figure 2A) and silenced (Figure 2B) *ho* in neurons, indicating a dual role of HO in the regulation of apoptosis in flies. What is interesting, old flies with elevated neuronal *ho* expression showed a significant reduction of *hid* mRNA, while old flies with silenced neuronal *ho* mRNA showed similar *hid* expression as the controls. These results imply that HO can be pro-apoptotic in young flies and anti-apoptotic in older flies, depending on its expression level in neurons.

Measurements of the apoptosis initiator caspase Dronc activity in different age groups (7- or 30-day-old) of flies with overexpressed or silenced *ho* in neurons confirmed the influence of *ho* expression on apoptosis (Figures 2C, D). In young flies, both overexpression and silencing of neuronal *ho* showed a significant increase in caspase Dronc activity in comparison to the parental control. Over time, caspase activity in flies with varying *ho* expression levels was still higher than in the parental control in 30-day-old flies. However, in the case of *elav > ho,* a significant decrease in caspase activity was found in comparing with young flies. Whereas, in *elav* > *hoRNAi*, caspase activity was higher in older flies. Notwithstanding, caspase Dronc activity in aging CS > *ho* or CS > *hoRNAi* did not show a similar trend of changes with age.

### 3.3 Age-dependent induction of autophagy-related genes by *ho* expression in neurons

**FIGURE 3:**
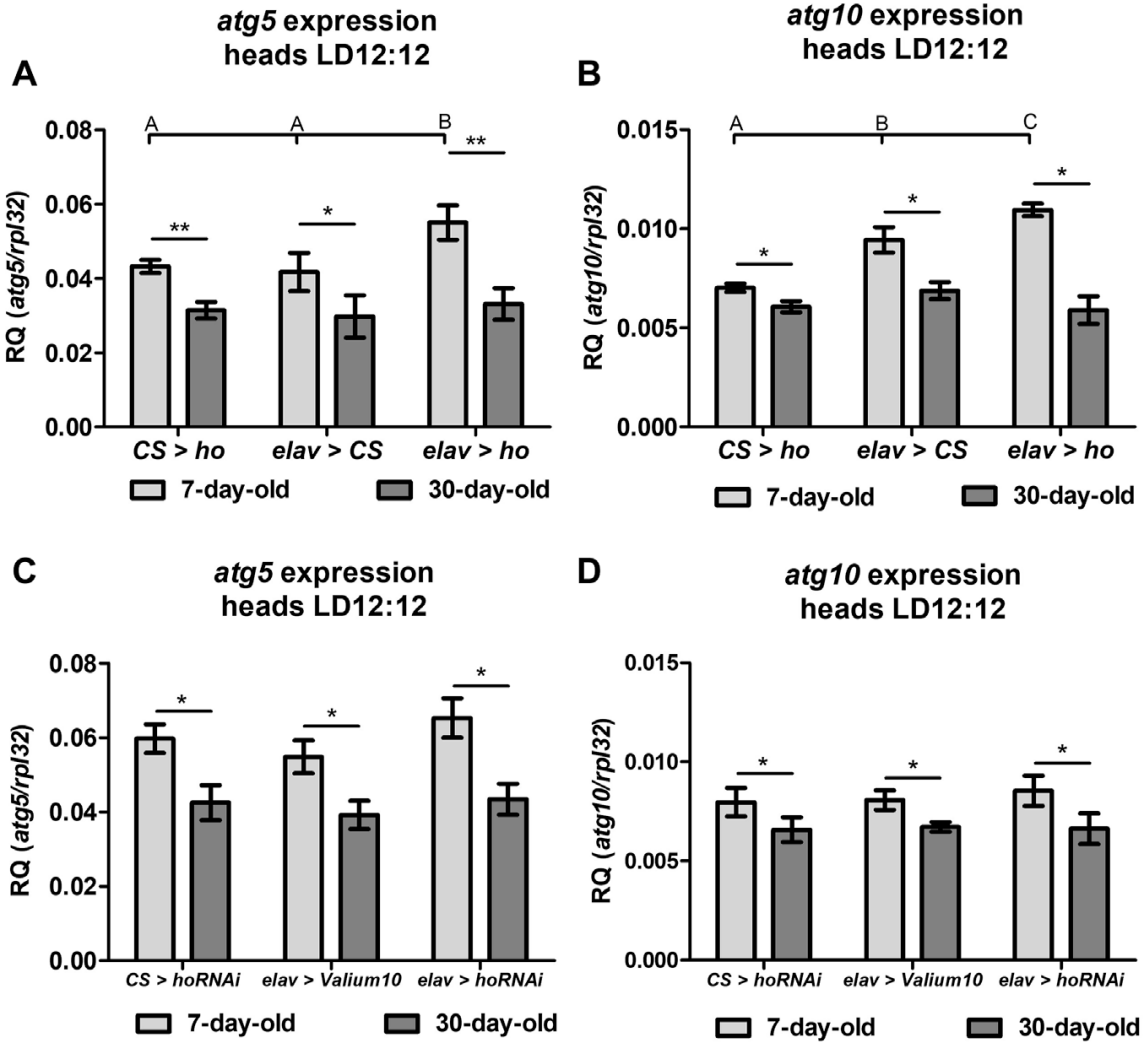
Expression of the *autophagy-related* genes (*atg5* and *atg10*) was induced in heads of flies with pan-neuronal *ho* overexpression (**(A)**–*atg5*, **(B)** *atg10*) but not with pan-neuronal *ho* silencing (**(C)**–*atg5*, **(D)** *atg10*) and was independent on age (means ± SD). Statistically significant differences between genotypes are indicated with different *letters* (non-parametric ANOVA Kruskal–Wallis and *post hoc* Conover-Iman tests: *atg5* in flies with *ho* overexpression and 7-day-old *p* = 0.02, 30-day-old *p* = 0.6; *atg10* in flies with *ho* overexpression in 7-day-old *p* = 0.003, 30-day-old *p* = 0.05; *atg5* in *ho* silencing in 7-day-old *p* = 0.1, 30-day-old *p =* 0.2; *atg10* in *ho* silencing in 7-day-old *p* = 0.5, 30-day-old *p* = 0.9) and between different age groups by *asterisks* (7-day-old vs. 30-day-old in each genotype; non-parametric Mann-Whitney test - *atg5*: *elav* > *ho p* = 0.01; CS > *ho p* = 0.008; *elav* > CS *p* = 0.03; *elav* > *hoRNAi p* = 0.03; CS > *hoRNAi p* = 0.02; *elav* > *Valium10 p* = 0.004; *atg10*: *elav* > *ho p* = 0.02; CS > *ho p* = 0.03; *elav* > CS *p* = 0.03; *elav* > *hoRNAi p* = 0.03; CS > *hoRNAi p* = 0.03; *elav* > *Valium10 p* = 0.02). These experiments were repeated at least three times per genotype or age group with 20 heads per repetition.

The expression level of autophagy-related genes (*atg5* and *atg10*) in fly heads with varying *ho* expression levels in neurons showed that autophagy significantly increased when *ho* expression was constantly upregulated, and this effect was only observed in young flies (Figures 3A, B). Regardless of age, the expression of *atg5* and *atg10* did not changes in flies with partially silenced *ho* expression in neurons (Figures 3C, D).

### 3.4 Cell-specific and age-dependent degenerations caused by *ho* expression

**FIGURE 4:**
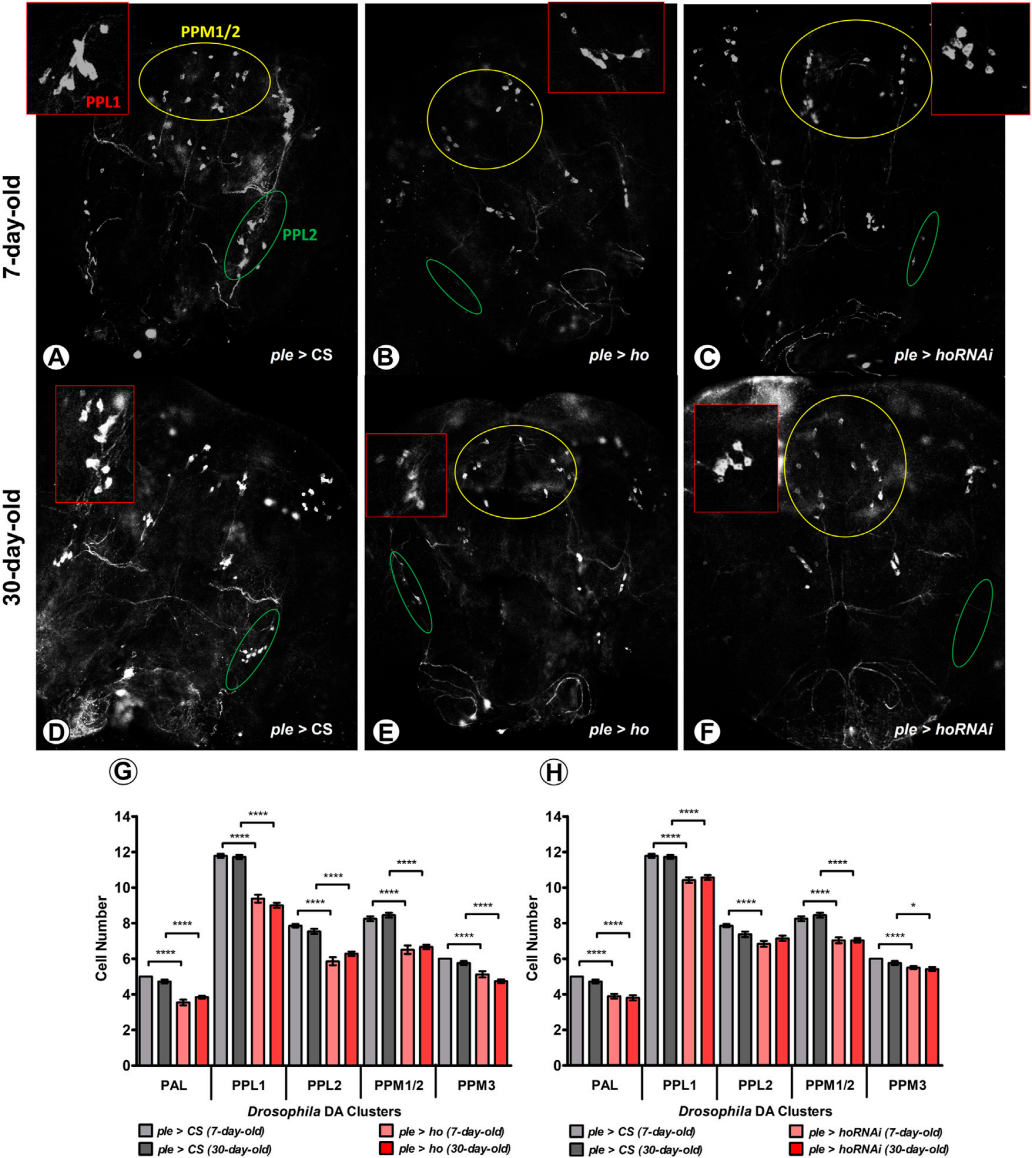
Increasing or decreasing the *ho* expression generates degeneration in DA cell clusters at an age-dependent manner. Dopaminergic (DA) neurons were particularly vulnerable to changes in *ho* expression levels and cell loss was higher in young flies **(A–C)** compared to older ones **(D–F)** in five different DA clusters. Statistically significant differences in DA cell number **(G,H)**, means ± SEM) between genotypes or different age groups are indicated with *asterisks* (7-day-old vs. 30-day-old in each genotype, analysed by parametric unpaired *t-*test with Welch’s correction between genotypes by age: *p ≤ 0.05, **p ≤ 0.01, ***p ≤ 0.001, ***p ≤ 0.0001). This immunostaining experiment was repeated at least three times for each genotype or age group with 10 brains per repetition. Exact *p* values for each DA cell cluster of the different combinations between genotypes and/or age groups are listed in Supplementary Table S1.

**FIGURE 5:**
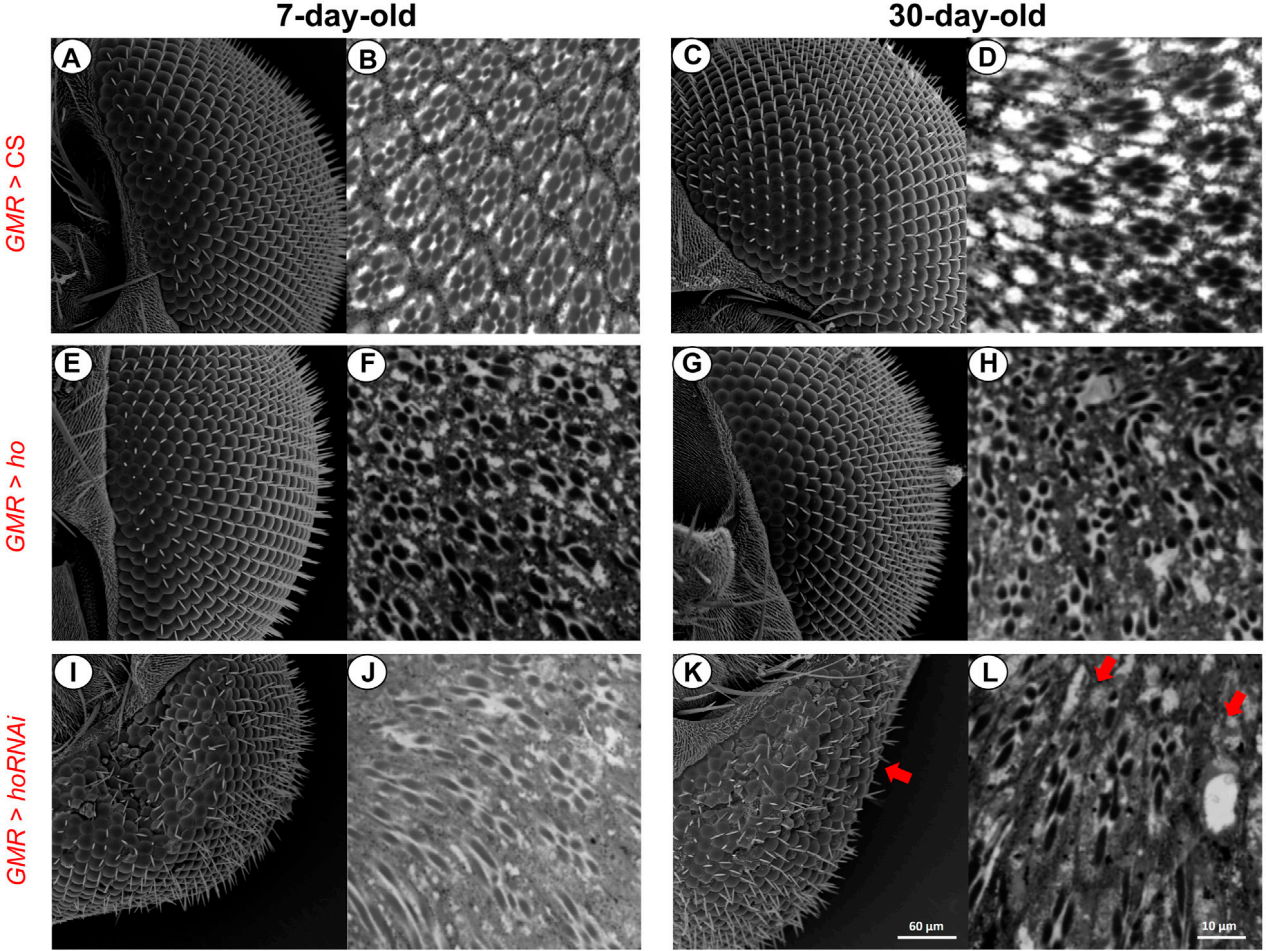
Retina photoreceptors were affected when the *ho* expression levels were changed (by overexpression or silencing) in the retina. Under normal conditions **(A–D)**, both ommatidia did not show any structural changes even with aging. After partially suppressing *ho* expression **(E–H)**, the eye morphology was changed and ommatidia were distorted regardless of age. Upregulation of *ho* in the retina photoreceptors did not produce any structural changes in the eye morphology **(I and K)** but in the proximal retina, degeneration was observed **(J and L)**.

Effects of apoptosis induction by HO were examined in dopaminergic (DA) neurons (Figure 4) and the retina photoreceptors (Figure 5). Maintaining *ho* expression at physiological level is especially important for DA cells since elevating or partially suppressing its expression level causes DA neuron degeneration (Figures 4A, D). In *ple* > *ho* and *ple* > *hoRNAi* strains there were observed abnormalities characterized by the disappearance of a part or whole DA neuron clusters (Figures 4B, C, E, F). Although this effect varied in intensity from one fly to another, drastic phenotypes were observed frequently, such as the complete degeneration of the PPL2 clusters. We quantified cell loss by counting neurons in each cluster (except the PAM cluster, in which the density of neurons is too high to allow precise numbering of the cells). The cell loss was observed in all dopaminergic clusters indiscriminately by varying *ho* expression levels in 7-day-old adult flies (Figures 4G, H; Supplementary Table S1). We did not find any further DA neuron degeneration in 30-day-old flies of the same genotypes.

In addition, when compared to the control (Figures 5A–D), *ho* overexpression in the fly’s retina photoreceptors did not cause any changes in the eye morphology even with aging (Figures 5E, G). However, we observed that retina ommatidia were slightly distorted in young adults and this was worsened with older flies (30-day-old) (Figures 5F, H). Moreover, reduced *ho* expression level causes severe disruptions of the eye morphology and internal structure of the retina (Figures 5I, J), which was the expected result supporting findings of other authors (Cui et al., 2008). Aging caused further degeneration of the retina (i.e., loss of photoreceptors in some ommatidia and their deformations which are pointed by arrows in Figures 5K, L).

### 3.5 Temperature-dependent effects of chronic curcumin supplementation on survival, climbing, and gene expression level

**FIGURE 6:**
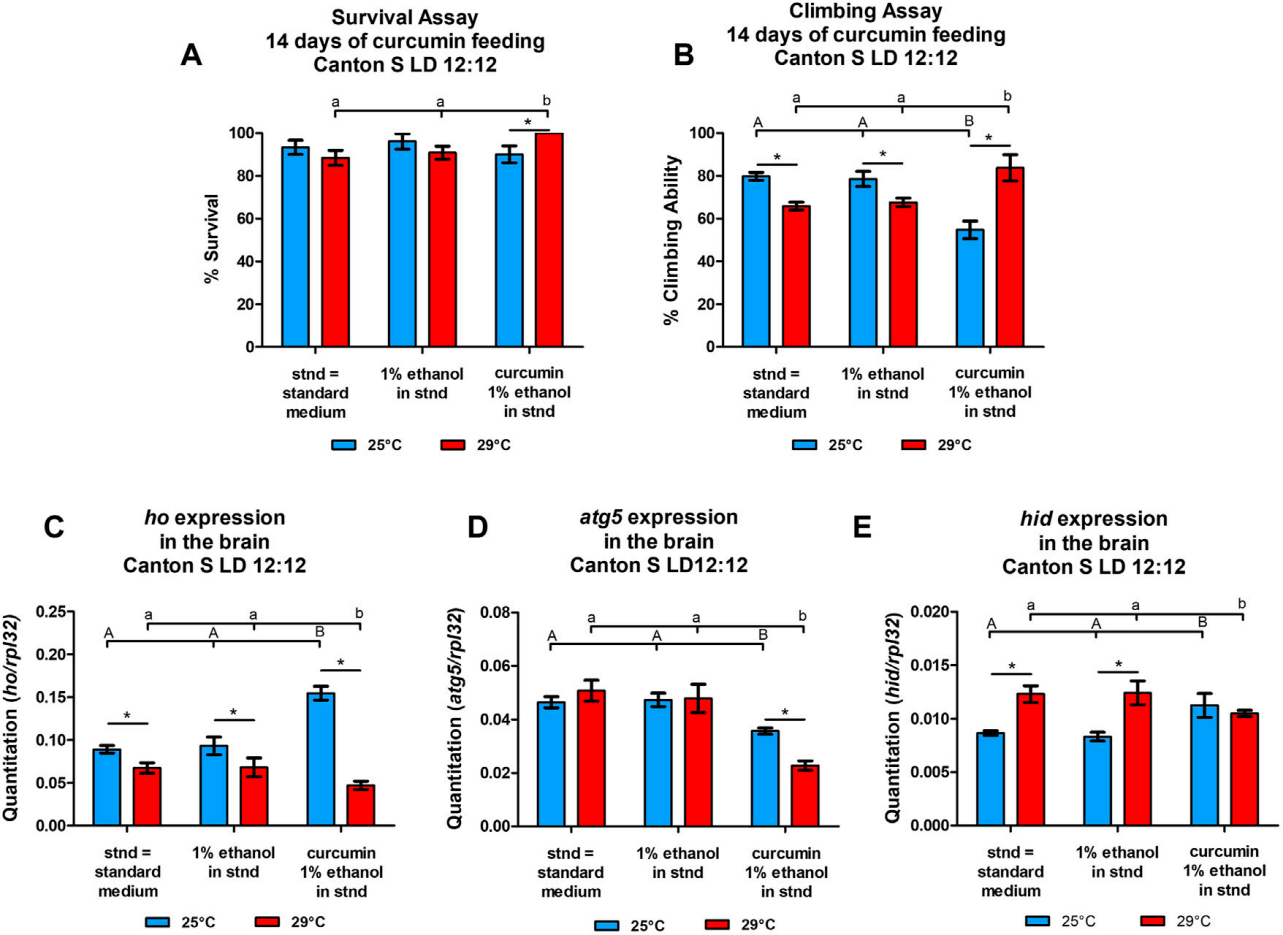
Temperature affects the survival **(A)**, climbing **(B)** (means ± SEM), and expression of *heme oxygenase* (*ho*, **(C)**, *autophagy-related gene 5* (*atg5*, **(D)**, and the cell death activator *head involution defective* (*hid*, **(E)** genes (means ± SD) after chronic supplementation of curcumin for 14°days. Statistically significant differences between groups are indicated with different *letters* (*capital letters* for 25°C and *small letters* for 29°C; non-parametric ANOVA–Kruskal Wallis and *post hoc* Conover-Iman tests; survival assay in 25°C *p* = 0.288, 29°C *p* = 0.046; climbing assay in 25°C *p* = 0.033, 29°C *p* = 0.025; parametric one-way ANOVA *F* test and Bonferroni multiple comparison test: *ho* mRNA level at 25°C *p* < 0.0001 & at 29°C *p* < 0.0001, *atg5* mRNA level at 25°C *p* < 0.0001 & at 29°C *p* < 0.0001, *hid* mRNA level at 25°C *p* < 0.0001 & at 29°C *p* < 0.0001) and between temperature conditions by *asterisks* (29°C vs. 25°C in each group; non-parametric Mann-Whitney test: survival assay: curcumin *p* = 0.032, 1% ethanol *p* = 0.200, and standard diet *p* = 0.268; climbing assay: curcumin *p* = 0.038, 1% ethanol *p* = 0.050, and standard diet *p* = 0.050; parametric unpaired *t-*test with Welch’s correction–*ho*: curcumin, 1% ethanol, and standard diet *p* < 0.0001; *atg5*: curcumin *p* < 0.0001, 1% ethanol *p* = 0.2117, standard diet *p* = 0.2029; *hid:* curcumin, 1% ethanol, and standard diet *p* < 0.0001). Both survival and climbing assays were repeated three times (30 flies in each treatment), while gene expression was studied in at least three repetitions for each treatment with 25 brains per repetition).

In the experiment of curcumin treatment for 14 days, we did not observe any differences in survival rate between experimental and control groups under normal temperature (25°C) (Figure 6A). However, at the same temperature, we found a significant reduction in climbing ability in CS flies supplemented with curcumin (Figure 6B). Surprisingly, under heat stress (29°C), flies supplemented with curcumin had higher survival and climbing ability compared to the controls. It was even improved comparing with flies fed with curcumin under normal temperature 25°C. Curcumin seemed to improve adult longevity and behavior despite high-temperature conditions.

The differences obtained in mRNA levels of *ho*, *atg5*, and *hid* between flies fed with curcumin and the controls maintained at two temperature regimes (25 or 29°C) showed that the effects of chronic curcumin supplementation change under high-temperature stress (Figures 6C–E). In the normal temperature (25 °C), curcumin significantly induced the expression of *ho* and *hid* in the fly’s brain in comparison to the group of flies fed with the standard diet. In the same condition, curcumin significantly decreased *atg5* expression. Flies fed with the standard diet and subjected to high-temperature stress showed cell death activation, decrease of *ho* mRNA, and unchanged *atg5* expression. After chronic supplementation with curcumin for 14 days under 29 °C, the *ho* transcript level was reduced, along with *atg5* mRNA, in comparing with two controls. Interestingly, *hid* expression after curcumin feeding under high-temperature stress remained at the same level but it was significantly lower than in both controls that were also maintained under high-temperature stress.

### 3.6 Effects of chronic curcumin supplementation on the survival, climbing ability, and expression of cell death activator gene in flies with overexpressed or silenced *ho* in neurons

The chronic supplementation of curcumin in transgenic flies with increased or decreased *ho* expression level in neurons generated various responses in flies’ survival and climbing ability*.*

**FIGURE 7:**
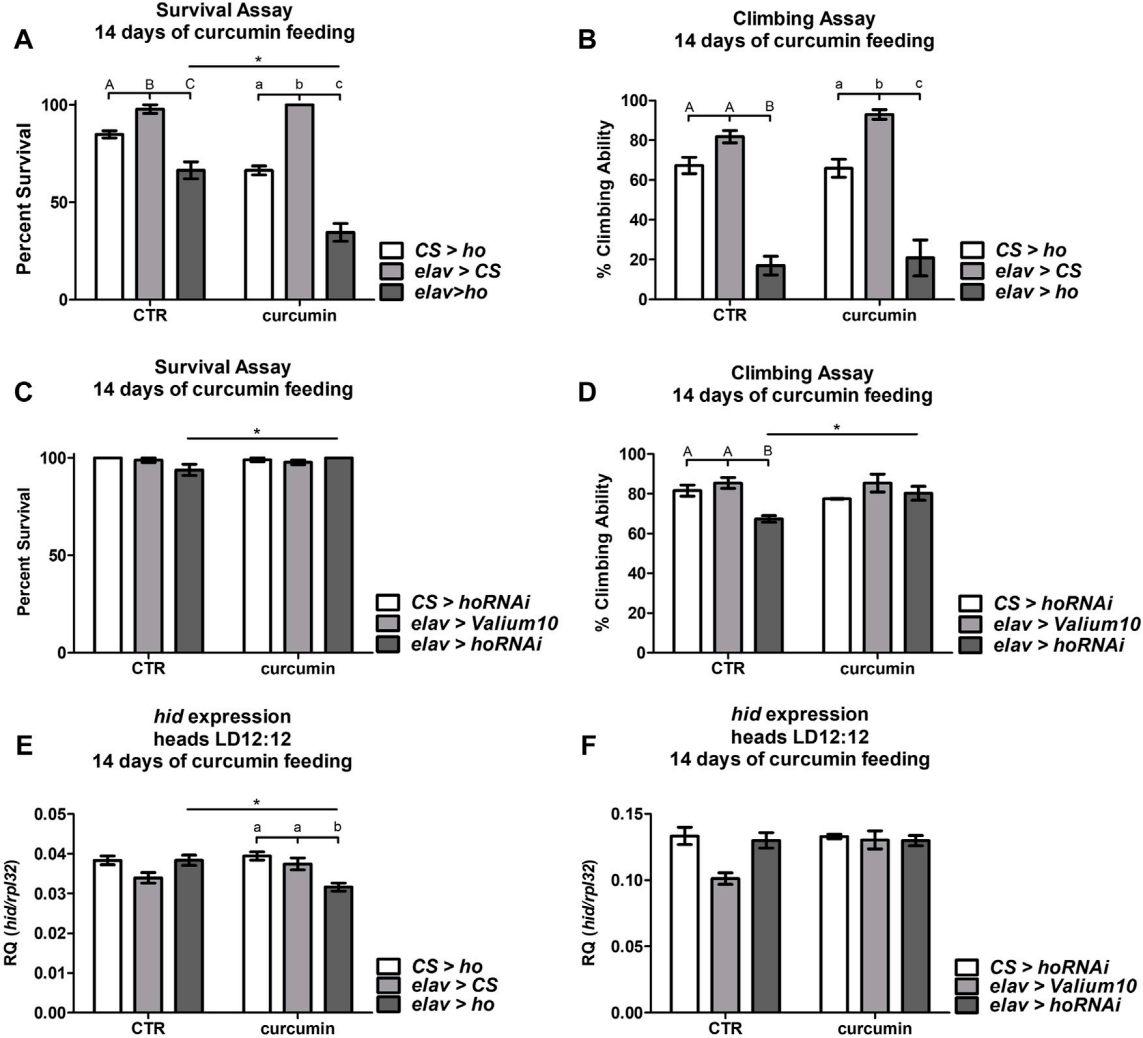
Chronic curcumin supplementation changed the survival, climbing ability, and expression of cell death activator gene *hid* in flies with overexpressed **(A,B)**, and **(E)** or silenced **(C,D)**, and **(F)** *ho* in all neurons. Statistically significant differences between genotypes per treatment are indicated with different *letters* (*capital letters* for control and *small letters* for groups supplemented with curcumin; non-parametric ANOVA Kruskal–Wallis and *post hoc* Conover-Iman tests) and between treated or non-treated flies with curcumin in each genotype by *asterisks* (non-parametric Mann Whitney test). Exact *p* values of percentage (%) of survival and climbing ability, including *hid* expression, of the different combinations between genotypes and treated or non-treated groups are listed in Supplementary Table S2.

Curcumin supplementation for 14 days was sufficient to reduce the survival of *elav* > *ho* flies when compared with the control groups and with those *elav* > *ho* flies that were not fed with curcumin (Figure 7A). In contrast, the climbing ability was lower in the experimental groups compared with their respective controls (Figure 7B). Nevertheless, this effect was independent of curcumin supplementation.

In *elav* > *hoRNAi* flies, we did not find any differences in survival with or without curcumin feeding (Figure 7C), however, survival was better in *elav* > *hoRNAi* supplemented with curcumin. The climbing behavior of flies with pan-neuronal *ho* silencing was rescued after chronic curcumin supplementation (Figure 7D). Nevertheless, this slight improvement in climbing ability of *elav* > *hoRNAi* fed with curcumin leveled off with the parental controls, which were not affected by curcumin.

To understand the effects of chronic curcumin supplementation in *elav* > *ho* and *elav* > *hoRNAi* strains, expression of the apoptotic gene *hid* was also quantified 14 days after exposing flies to this exogenous antioxidant. The reduction in survival and climbing percentages in *elav* > *ho* flies that were fed with curcumin was associated with significant decline of *hid* expression (Figure 7E). Surprisingly, the mRNA level of *hid* remained unchanged in flies with partially silenced *ho* in neurons even after 14 days of curcumin supplementation (Figure 7F). This result indicates that the pro-apoptotic effect of curcumin is under the control of HO.

## 4 Discussion

The obtained results showed that neuronal HO in *Drosophila* should be maintained at an adequate physiological level because if it is disrupted, it leads to apoptosis, a programmed cell death. Upregulation of apoptosis by excessive level of HO affects many cellular processes, which may be deleterious leading to neuron cell death. If left uncontrolled, it is possible that apoptosis can further facilitate the development of neurodegenerative diseases (Cui et al., 2021; Erekat, 2022).

The effects that we initially observed with higher or lower than normal *ho* expression levels were early deaths and locomotion defects, which were more prevalent in flies with continuous pan-neuronal *ho* overexpression*.* In contrast, flies with continuous pan-neuronal *ho* silencing improved over time and were not different from their parental controls. They also lived longer than those with *ho* overexpression. Next, we investigated the involvement of *ho* in the regulation of apoptosis to explain the observed survival and climbing changes. We found that HO has a dual role in the regulation of apoptosis. The upregulation of pro-apoptotic gene *hid* in neurons was detected in both *elav* > *ho* or *elav* > *hoRNAi* flies. In our previous study, this effect was also observed and was dependent on the time of the day (Abaquita et al., 2021). When changes of *ho* expression were directed to dopaminergic (DA) neurons, both *ple* > *ho* and *ple* > *hoRNAi* flies showed significant degeneration of DA cell clusters (PAL, PPL1, PPL2, PPM1/2, and PPM3) in the early stage of adult life, which we expected to occur when apoptosis is dysregulated by varying *ho* expression level. It was also correlated with the increase of caspase Dronc (mammalian ortholog of caspase-9) activity in 7-day-old *elav* > *ho* or *elav* > *hoRNAi* flies. Under normal conditions, apoptosis remains untriggered in the early phases of adult life and can increase after stress (Miyoshi et al., 2006; Jahnke and Bedi, 2007). These variations of *ho* expression levels in neurons can be observed in flies that are under stress. However, cells of young individuals are more resistant to cell death activation caused by unfavorable conditions (Miyoshi et al., 2006).

This two-sided effect of HO in regulating apoptosis changes during aging. We observed a gradual decrease of *hid* expression in strains with *ho* overexpression and normal *hid* mRNA level in strains with *ho* silencing with aging, It has been supported by the lack of changes in DA cell numbers regardless of the *ho* expression level. Nevertheless, the caspase Dronc activity in older transgenic flies with either *ho* overexpression or silencing in neurons, was higher compared with the control. It has been reported that caspase-9 (mammalian counterpart of caspase Dronc) activity increases in aging brains, which have a lower (<20%) number of dying cells when compared to younger brains and this is accompanied by decreased expression of pro-caspase-3. It has been suggested as a survival strategy for aging neurons, which can no longer regenerate, but in which the central apoptotic machinery downstream of caspase-9 is inactivated (Ohsawa et al., 2009). Moreover, apoptosis may not be frequent during aging because cells produce more anti-apoptotic proteins than pro-apoptotic ones, which is why it is difficult to force post-mitotic and senescent cells to apoptosis (Pollack et al., 2002) or cell degeneration (including DA neurons: White et al., 2010) under normal conditions, as depicted in our control groups. This was explained by the limitation of energy consumption to extend the lifespan (Pletcher et al., 2002). In turn low ATP level in cells of older animals increases their susceptibility to cell death (necrosis instead of apoptosis) under stress (Miyoshi et al., 2006). However, there are also reports that apoptosis may increase over time in flies (Zheng et al., 2005) and mammals (Zhang et al., 2003; Chung and Ng, 2006). The latter findings may indicate activation of cytoprotective processes by the increase of caspase activity which is accordingly possible in response to aging (Shalini et al., 2015; Tower, 2015).

We also observed that positive and negative regulation of apoptosis by HO is cell-specific. Lower or higher than normal expression of *ho* has strong effects on DA neurons. This group of neurons is particularly vulnerable to apoptosis under stressful conditions (Hirsch et al., 1988; Damier et al., 1999; Dauer and Przedborski, 2003; Greene et al., 2003; Coulom & Birman, 2004; Yang et al., 2006; Periquet et al., 2007; Wang and Michaelis, 2010; Doktór et al., 2019; Davis et al., 2021). The vulnerability of DA neurons is attributed to dopamine synthesis and its disruption leads to oxidative stress (Meiser et al., 2013). In mammals, enhanced dopamine synthesis leads to loss of neurons and decreases cytoprotection by reducing alpha-synuclein accumulation (Mori et al., 2006). In flies, HO activity in postsynaptic mushroom body neurons was reported to indirectly facilitate the non-canonical dopamine release (Ueno et al., 2020). Here, we showed that DA neurons require the balance in *ho* expression to avoid degeneration. Apart from DA neurons, there are also other neuronal populations vulnerable to stress (Hyman et al., 1984; Braak & Braak, 1991; Terry et al., 1991; Rowland & Shneider, 2001; Wang and Michaelis, 2010), including retinal cells (Damulewicz et al., 2017a; Damulewicz et al., 2017b). In the present study, we found a different response in photoreceptors than in DA neurons. We observed that the retina photoreceptor pattern was distorted in strains with lower or higher *ho* expression than physiological one, while the eye morphology was changed only after *ho* silencing because HO controls expression of many genes (Damulewicz et al., 2019).

The experiments with curcumin as an antioxidant and a compound affecting HO and apoptosis revealed interactions between HO signaling pathway and apoptosis. It has already been reported in both mammals (McNally et al., 2007; Lima et al., 2011; Cremers et al., 2014; Masuelli et al., 2017; Yang et al., 2017; Peng et al., 2018; Zhang et al., 2018; Zhong et al., 2018) and flies (Abaquita et al., 2021). Curcumin activates the nuclear factor erythroid 2-like 2 (Nrf2) pathway that regulates *ho* expression (Peng et al., 2018; Zhong et al., 2018). Curcumin also induces endoplasmic reticulum stress, calcium release, and destabilization of mitochondria, which result in apoptosis (Moustapha et al., 2015) after blocking autophagy (Masuelli et al., 2017). Despite that, curcumin has been shown to inhibit apoptosis in the fly’s brain (Siddique et al., 2014), and to induce autophagy (Moustapha et al., 2015). Apoptosis- and HO-inducing effects of curcumin changed when we applied high-temperature stress which antagonized both HO and apoptosis. In control flies, the expression of apoptotic gene *hid* was significantly higher under heat stress, while *ho* mRNA level was lower compared to those in normal temperature conditions. This can be linked to the reduction of *Drosophila* energy stores under high-temperature stress, which disrupts the partitioning of energy, obtained from food, into various biological processes (Klepsatel et al., 2016; 2019). Heat shock also speeds up the metabolic rate in flies (Mołoń et al., 2020) and typically increases apoptosis since temperature stress can damage cells and cause their death (Bellmann et al., 2010). Notwithstanding, flies supplemented with curcumin can still survive and even have an extended lifespan under heat stress. By increasing the expression of superoxide dismutase (SOD), catalase (CAT), and phospholipid hydroperoxide glutathione peroxidase (PHGPx) and decreasing the expression of HSP70 and HSP83, curcumin palliated oxidative stress caused by high-temperature stress (Chen et al., 2018). Our results obtained in this study support this finding as we found better survival and climbing behavior in flies chronically fed with curcumin during exposure to heat stress.

Our study proved that excessive HO can be very detrimental since upregulating (by genetic manipulations and chronic curcumin supplementation at the same time) HO strongly affects the survival of flies. We found that in this state, apoptosis was reduced just like in old, non-supplemented curcumin flies. The combination of both was cytotoxic, instead of cytoprotective. It has been reported that increasing the recommended doses of antioxidative substances like curcumin is toxic and can negatively affect pro-survival mechanisms (Phom et al., 2014). On the other hand, chronic curcumin feeding in flies with silenced neuronal *ho* expression exerted better survival rates and climbing behavior. It suggests that curcumin-induced *ho* expression mediates the pro-apoptotic effect of curcumin. Our findings, therefore, support a partial inhibition of HO as a therapeutic approach in curcumin-dependent medical treatments against age-related and neurodegenerative diseases. In some mammalian studies it have been shown that pharmacological inhibition of HO could be a promising supplementary therapeutic approach for age-related diseases (Marinissen et al., 2006; Frank et al., 2007; Hirai et al., 2007; Mayerhofer et al., 2008; Nowis et al., 2008; Sass et al., 2008; Shi & Fang, 2008; Costa et al., 2016; Podkalicka et al., 2018; Yang et al., 2018; Luu Hoang et al., 2021). According to our data, this particular approach can be applied at a certain age to optimize its potential. The opposite effect was observed in the case of *ho* overexpressed in neurons since flies, at the early stage of adult life, showed a phenotype that is observed in old flies. However, these results might be cell type dependent since there are also conflicting reports about HO-1 upregulation which inhibits apoptosis (Ryter, 2021).

There are many possible explanations why constant upregulation of HO is dangerous because of: i) promotion of the oxidation of cholesterol to oxysterols (Vaya et al., 2007; Vaya and Schipper, 2007); ii) high-glucose-derived oxidative stress by MAPK-mediated NF-κB and AP-1 cascades (Hsieh et al., 2010); iii) accumulation of reaction products at critical levels (Wu and Hsieh, 2022), particularly iron overload or ferroptosis (Suttner & Dennery, 1999; Puntarulo, 2005; Li et al., 2012; Fibach and Rachmilewitz, 2017); and lastly, iv) translocation into non-endoplasmic reticulum compartments (Lin et al., 2007; Sacca et al., 2007; Bindu et al., 2011; Bolisetty et al., 2013; Bansal et al., 2014; Biswas et al., 2014; Dunn et al., 2014; Tibullo et al., 2016; Mascaró et al., 2021; Yang and Wang, 2022). Furthermore, the active-site structure of dHO is quite different from mammalian HOs, even though it acts as a real HO (Zhang et al., 2004). Hence, there is a need for further studies of the HO pathway in flies from a mechanistic and structural perspective in order to find missing key regulatory elements of its pro-apoptotic activity. We found that the pro-apoptotic role of HO upregulation at the early stage of adult life was accompanied by autophagy induction. It would be interesting to further check pro-survival mechanisms activated by *ho* silencing in neurons of flies exposed to oxidative stress.

In conclusion, our study showed that it is important to keep a balance in HO regulation in cells as we showed that changes in neuronal *ho* expression level can promote cell death and may disrupt homeostasis. The excessive concentration of HO can negatively affect the pro-survival processes and its deficiency can be supplemented with antioxidant substances like curcumin. In addition, the dual role of HO in regulating apoptosis depends on many factors including the *ho* expression level, age, and cell type. There is a need for a careful selection of antioxidant supplementation as a medical treatment or a diet since it may complicate or produce negative effects, instead of positive ones.

## Data Availability

The raw data supporting the conclusions of this article will be made available by the authors, without undue reservation.
