# Supplementary material for: The dual role of heme oxygenase in regulating apoptosis in the nervous system of Drosophila melanogaster
Source: Front Physiol. 2023 Feb 13;14:1060175. doi: 10.3389/fphys.2023.1060175 (PMC9969482; doi:10.3389/fphys.2023.1060175)
Supplement: Supplementary file 1 [file DataSheet1.pdf]

Supplementary Material

Supplementary Figure

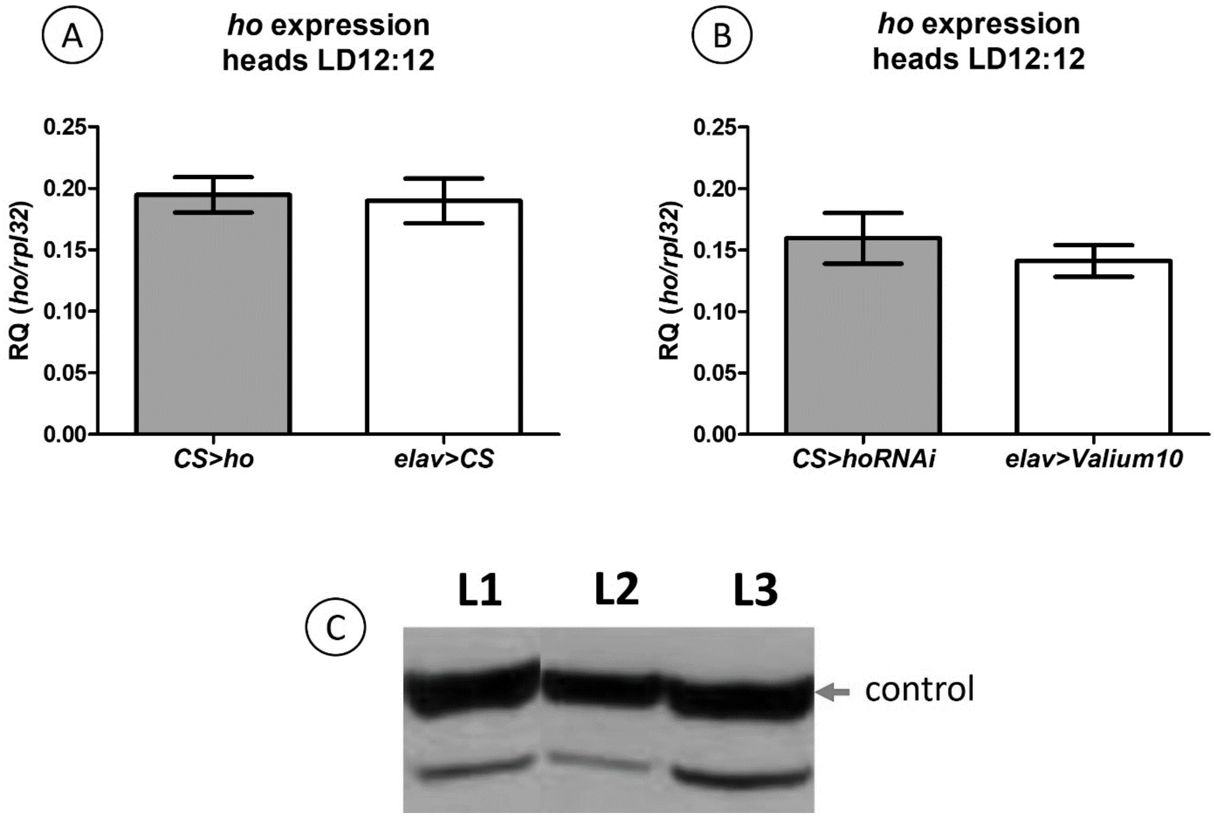

Supplementary Figure 1. The expression of the *ho* gene did not change between control groups (A-B). Protein levels were also confirmed to be overexpressed or silenced in the experimental flies (C) with the following ratio: CS (L1) = 0.282, *elav>hoRNAi* (L2) = 0.167, and *elav>ho* (L3) = 0.445.

Supplementary Tables

Supplementary Table 1. Exact *p* values for each dopaminergic neuron (DA) cluster of the different combinations between genotypes and/or age groups.

7- vs 30-day-old

Genotype	DA cluster	*p*	Genotype	DA cluster	*p*	Genotype	DA cluster	*p*
ple>ho	PAL	0.2	*ple> hoRNAi*	PAL	0.2	*ple*>CS	PAL	0.2
	PPL1	0.3		PPL1	1.0		PPL1	0.4
	PPL2	0.2		PPL2	0.4		PPL2	0.2
	PPM1/2	0.7		PPM1/2	0.8		PPM1/2	0.6
	PPM3	0.2		PPM3	1.0		PPM3	0.1

***ple>ho* vs *ple*>CS (7-day-old)**		***ple>ho* vs *ple*>CS (30-day-old)**	
DA cluster	*p*	DA cluster	*p*
PAL	< 0.0001	PAL	< 0.0001
PPL1	< 0.0001	PPL1	< 0.0001
PPL2	< 0.0001	PPL2	< 0.0001
PPM1/2	< 0.0001	PPM1/2	< 0.0001
PPM3	< 0.0001	PPM3	< 0.0001

***ple>hoRNAi* vs *ple*>CS (7-day-old)**		***ple>hoRNAi* vs *ple*>CS (30-day-old)**	
DA cluster	*p*	DA cluster	*p*
PAL	< 0.0001	PAL	< 0.0001
PPL1	< 0.0001	PPL1	< 0.0001
PPL2	< 0.0001	PPL2	0.3
PPM1/2	< 0.0001	PPM1/2	< 0.0001
PPM3	< 0.0001	PPM3	0.03

Supplementary Table 2. Exact *p* values of the percentage of survival and climbing ability, including *hid* expression, of the different combinations between genotypes and treated or non-treated groups. The non-parametric ANOVA Kruskal-Wallis test was used to analyse each combination.

**% survival**					
Genotype	Treatment	*p*	Genotype	Treatment	*p*
elav>ho vs *elav*>CS vs CS>*ho*	CTR	0.02	*elav>hoRNAi* vs *elav>Valium10* vs CS>*hoRNAi*	CTR	0.02
	CUR	0.003		CUR	0.03
**% climbing ability**					
Genotype	Treatment	*p*	Genotype	Treatment	*p*
elav>ho vs *elav*>CS vs CS>*ho*	CTR	0.1	*elav>hoRNAi* vs *elav>Valium10* vs CS>*hoRNAi*	CTR	0.05
	CUR	0.2		CUR	0.6
***hid* expression**					
Genotype	Treatment	*p*	Genotype	Treatment	*p*
elav>ho vs *elav*>CS vs CS>*ho*	CTR	0.07	*elav>hoRNAi* vs *elav>Valium10* vs CS>*hoRNAi*	CTR	0.02*
	CUR	0.01		CUR	0.8

*inconsistent significant result (one parental control is not different from the experimental group)
